# Supplementary material for: Implementation barriers and facilitators for referral from the hospital to community-based lifestyle interventions from the perspective of lifestyle professionals: A qualitative study
Source: PLoS One. 2024 Jun 27;19(6):e0304053. doi: 10.1371/journal.pone.0304053 (PMC11210764; doi:10.1371/journal.pone.0304053)
Supplement: S1 Appendix — (PDF) [file pone.0304053.s001.pdf]

S1 Appendix. **Topic Guide for interview stakeholders: CBLI**

<table>
<tr><td colspan="2">Introduction<br>Today, we would like to discuss topics concerning the development of an LFO in the hospital. The aim of the LOFIT-study is to develop, implement and evaluate the LFO within the hospitals Amsterdam UMC and UMC Groningen. AN LFO is a place in the hospital where patients are guided by a lifestyle broker to find adequate community based lifestyle interventions. With lifestyle we mean behavior that patients have developed concerning healthy eating, smoking, physical activity, excessive alcohol intake, stress and sleep. The aim of the LOFIT-study is to optimize hospital care by making the referral to lifestyle guidance in the community an integral part of usual care. This interview will add to the scientific foundation of the LFO as well as the development and the implementation.</td></tr>
<tr><td colspan="2">Demographic information<br>- Can you tell something about your professional background and the duration of your employment in this line of work?<br>Use the provided information to complete the table below.<br><br>If the respondent's job is lifestyle coach, proceed to parts 1, 2 and 5 now.<br>If the respondent's job is care sport connector, ask the following question and proceed as stated below.<br>- Which tasks/responsibilities do you have in your job? (executing lifestyle interventions and/or connector between clients and organizations and/or other duties?)<br>Choose:<br>➔ respondent is both connector and executor of interventions: proceed to parts 1-5<br>➔ respondent is solely an executor of interventions: proceed to parts 1,2 and 5<br>➔ respondent is solely a connector: proceed to parts 3, 4 and 5<br><br>
<table>
<tr><td>Question</td><td>Response</td></tr>
<tr><td>Age</td><td>… year</td></tr>
<tr><td>Gender</td><td>Man/woman/other</td></tr>
<tr><td>(care) Institution</td><td></td></tr>
<tr><td>Job title</td><td>*</td></tr>
<tr><td>Years of experience in current job</td><td>… year</td></tr>
<tr><td>Type of patient groups treated mostly</td><td></td></tr>
</table></td></tr>
<tr><td>Domain</td><td>Questions and Probe</td></tr>
<tr><td>Part 1<br><br>Lifestyle intervention</td><td>- Can you tell us more about the lifestyle interventions you offer to patients/are offered in your organization?<br>o Prompt: where is it offered? (i.e. which region in the Netherlands)<br>o Prompt: for which target group is it suitable? And why this specific group(s)? [specific CVD or osteoarthritis?]<br>o Prompt: which lifestyle behaviors are targeted?<br>o Prompt: what information is given to the patient? (i.e. which advice, or referring to guidelines and standards)<br>o Prompt: how and how long will participants/patients be guided? And what happens afterwards?</td></tr>
</table>

| | |
|---|---|
| | o Prompt: Which financial agreements are made? (i.e. agreements with insurance companies (which); contribution patient?)<br>o Prompt: what do you do to guarantee  the quality of the professionals?<br>o Prompt: How does your intervention relate to other lifestyle interventions?<br>o Prompt: what is your current experience with referral from primary care (general practitioner) and secondary care (hospital) to your lifestyle intervention? |
| **Part 2**<br><br>Experiences with effects of the lifestyle intervention | - What is your current experience with patients that participated in "*name lifestyle intervention*"?<br>o Prompt: patients' motivation for making lifestyle changes<br>o Prompt: actual lifestyle changes that patients made.<br>o Prompt: maintaining long-term lifestyle changes made by patients<br>o Prompt: effects on patient's disease (e.g. reduction of medication use, reduction of complications) |
| **Part 3**<br><br>Connecting partners | - To which lifestyle interventions do you refer?<br>o Why these interventions?<br>• Do you work together regularly?<br>• Based on what do you refer to these interventions?<br>o Do you monitor progress of the referred client?<br>• How often?<br>• How? Are you satisfied with this manner? |
| **Part 4**<br><br>Role of care sport connector | - What are your thoughts about the role that a care sport connector could have in the referral from the LFO in the hospital, to lifestyle interventions in the community?<br>o What do you think of the idea of a care sport connector having a central and coordinating role in this process?<br>o What are facilitating factors?<br>o What are possible hindering factors?<br>o Who do you think should be responsible for providing feedback to the LFO? |
| **Part 5**<br><br>Preferences and needs regarding collaboration with the LFO in the hospital | *Previous research shows that physicians in the hospital have too little time to adequately discuss a patients' lifestyle. That is why we want to develop an LFO in the hospital, where experts have time and knowledge to discuss lifestyle and behavioral change but also have knowledge about possible referral options in the community, in order to guide the patient optimally in changing their behavior of nutrition, smoking, physical activity, stress, sleep and alcohol intake. We would like to use your suggestions to further develop and implement such a referral system.*<br><br>- What are your thoughts about how we can refer patients from the LFO to your intervention?<br>o Prompt: what would be helping and what could be a barrier? |

| | |
|---|---|
| | o Prompt: who should make the initial appointment with you? Lifestyle broker or patient?<br>o Prompt: what is the best way to make an appointment with you? (by telephone, via e-mail/other)<br>o Prompt: would you like to receive information about the patient with the referral? And if yes, which type of information? And how would you like to receive this information? (digitally /face-te-face)?<br><br>*We feel it is important to provide feedback about the patients' progress to the referring physician. Therefore, we would like the LFO to be kept informed .*<br>- What are your thoughts about the best way to organize this feedback and to align this with your normal workflow?<br>o Prompt: how would you like to provide this feedback (digitally – face to face)?<br>o Prompt: what would be the content of such feedback?<br>o Prompt: how often would it be realistic to provide such feedback? |
| *Closing*<br><br>*Summarize all preferences and needs by the respondent.*<br>- Do you have any comments or do you want to add anything that you think is relevant for this interview?<br>- Thank you very much for your participation. The results will be used to develop and implement the LFO.<br>- Do you have any recommendations of other colleagues that might be interested?<br>- Would you like to be kept informed regarding the progress of this project? For instance, would you like to receive the newsletter? | |
