# Supplementary material for: Implementation barriers and facilitators for referral from the hospital to community-based lifestyle interventions from the perspective of lifestyle professionals: A qualitative study
Source: PLoS One. 2024 Jun 27;19(6):e0304053. doi: 10.1371/journal.pone.0304053 (PMC11210764; doi:10.1371/journal.pone.0304053)
Supplement: S2 Appendix — (PDF) [file pone.0304053.s002.pdf]

S2 Appendix. **Codebook.**

**Domain**	**Code**	**Description**
Suggestions	LFO (LFO)	Any suggestions on improving the LFO in helping patients improving their health through lifestyle changes
Suggestions	Community based lifestyle interventions	Any comments/ideas of improving the knowledge/skills of community based lifestyle interventions (CBLI)
Suggestions	Policy	Any comments about national policy that could influence our project (+/-)
Suggestions	Follow-up research	Any comments on participating in follow up research (+/-)
Referral LFO (LFO) to community based lifestyle intervention (CBLI)	Referral content (+)	Referral information which can help community based lifestyle interventions improve care , such as provision of patient profile, patient lifestyle goals, relevant life events, relevant social status and expected investment of the CBLI of choice (+)
Referral LFO (LFO) to community based lifestyle intervention (CBLI)	Referral content (-)	Referral information that impairs community based lifestyle intervention in provision of care, such as unable to form own opinion because of given information, lack of necessary information, not providing accurate information about CBLI (-)
Referral LFO (LFO) to community based lifestyle intervention (CBLI)	Knowledge referral options (+)	All factors improving knowledge of the lifestyle broker on referral options, such as the use of certain networks, databases or knowledge on interventions
Referral LFO (LFO) to community based lifestyle intervention (CBLI)	Knowledge referral options (-)	There are a lot of interventions in the Netherlands and it is difficult for lifestyle broker (LB) to know them all (-). Some interventions are not easy to find (-)
Referral LFO (LFO) to community based lifestyle intervention (CBLI)	Accessibility lifestyle interventions (+)	All comments about measures that make lifestyle interventions better accessible for the patient, for instance funding and availability(+)

Referral LFO (LFO) to community based lifestyle intervention (CBLI)	Accessibility lifestyle interventions (-)	All factors that make lifestyle interventions less accessible for patients, for instance financial limitations or in smaller towns, there is limited availability of interventions (-)
Referral LFO (LFO) to community based lifestyle intervention (CBLI)	Intermediary referral option (+)	All codes in favor of using intermediaries as referral options (such as care sport connectors or nurse practitioners (POH-er)). (+)
Referral LFO (LFO) to community based lifestyle intervention (CBLI)	Intermediary referral option (-)	All codes that describe factors that impair the use of an intermediary referral option, such as the variety in job title of care sport connectors or the lack of time for extra work (-)
Referral LFO (LFO) to community based lifestyle intervention (CBLI)	Transfer to intermediary or CBLI (+)	All factors that facilitate the patient to follow through to the CBLI, such as initiation of appointment by lifestyle intervention or lifestyle broker (+)
Referral LFO (LFO) to community based lifestyle intervention (CBLI)	Transfer to intermediary or CBLI (-)	All factors that impair the patient to follow through to the CBLI, such as ….(loss of motivation, anxiety, …)
Feedback from CBLI to LFO	Feedback process (+)	All process factors that facilitate CBLI to report to the LFO about the client's progress, such as available time, feedback routes. Do not code digital facilitators in this code; these should be coded as Digital facilities (+) in domain Collaboration
Feedback from CBLI to LFO	Feedback process (-)	All process factors that impair CBLI to report to the LFO about the client's progress, such as available time, feedback routes. Do not code digital barriers in this code; these should be coded as Digital facilities (-) in domain Collaboration

Feedback from CBLI to LFO	Feedback content (+)	All factors that facilitate the LFO to keep track of lifestyle progress of patient regarding the content of the feedback
Feedback from CBLI to LFO	Feedback content (-)	All factors that impair the LFO to keep track of lifestyle progress of patient regarding the content of the feedback
Collaboration between LFO and CBLI	Digital facilities (+)	Factors that facilitate digital communication (+) from LFO to CBLI and back related to digital system, security of data
Collaboration between LFO and CBLI	Digital facilities (-)	Factors that impair digital communication (-) from LFO to CBLI and back related to digital system, security of data
Collaboration between LFO and CBLI	Partnership (+)	All relational factors that help building a good connection between the LFO and CBLI (+), such as personal acquaintance, recognition of the other party and mutual trust, wanting to act as a team. Comments on the practical allocation of tasks should be coded as Harmonization (+)
Collaboration between LFO and CBLI	Partnership (-)	All relational factors that impairs a good connection between the LFO and CBLI, such as not knowing each other personally, not trusting each other, not feeling appreciated(-). Comments on the practical allocation of tasks should be coded as Harmonization (-)
Collaboration between LFO and CBLI	Harmonization (+)	All comments that facilitate working together efficiently, like aligning tasks that need to be performed by LB and CBLI<br>Comments about which task should be performed by whom, when and how often. When comments are *needed* for efficient collaboration they should be coded as Harmonization (+); comments that are *mere practical suggestions* for a better LFO should be coded as 'suggestions LFO'.
Collaboration between LFO and CBLI	Harmonization (-)	All comments that impair working together efficiently at a practical level, like having unjust or unclear expectations of each other or no clear allocation of tasks. Code relational factors regarding personal acquaintance, trust and appreciation as 'partnership (+/-)'

Barriers experienced by community professionals		Barriers that community professionals experience in relation to their job that can have effect on the performance of the LFO.<br>Note: these barriers are not directly related to the LFO, but the consequences could have a negative effect on the LFO.
Context	Regional collaboration	All comments about professional collaboration in the community, how it is performed, how often, with whom.
Context	Role and tasks of the organization	Comments about the role and tasks of the organization
Context	Education & work experience professional	Comments about the education and relevant work experience of the professionals in the community
Context	Intervention target group	Comments about the target group(s) of the intervention
Context	Intervention objective	Comments about the goal(s) of the intervention or the goals of the participant
Context	Intervention duration	Comments about the duration and the frequency of the intervention
Context	Intervention content	Comments about the content of the intervention
Context	Intervention Results	Comments about the results of the intervention. Changes that professionals notice in their clients or that clients talk about.
Context	Intervention foundation	Comments about the underlying concept of the intervention, such as a theory or method.
