# Supplementary material for: Implementation barriers and facilitators for referral from the hospital to community-based lifestyle interventions from the perspective of lifestyle professionals: A qualitative study
Source: PLoS One. 2024 Jun 27;19(6):e0304053. doi: 10.1371/journal.pone.0304053 (PMC11210764; doi:10.1371/journal.pone.0304053)
Supplement: S3 Appendix — B = barrier; F = facilitator. (PDF) [file pone.0304053.s003.pdf]

S3 Appendix. **Summary of all barriers and facilitators per theme and subtheme(s).** B= barrier, F= facilitator

Theme	Subtheme 1	Subtheme 2	B/F	Description
Referral options from the LFO	Accessibility of referral options	Financial accessibility	B	Lifestyle interventions can be too expensive for patients with low income
			B	People with low SES are not used to spend money on wellbeing
			B	Health care insurance coverage is not enough for needed (nutritional)care
			F	Combined lifestyle intervention is financially fully covered by health care insurance
			F	Municipalities provide funding opportunities
			B	Applying for funding is difficult for some patients
Referral options from the LFO	Accessibility of referral options	Cultural accessibility	B	Few referral options for people who do not speak English or Dutch
			B	Combined lifestyle intervention offered almost exclusively in Dutch language
			B	It is hard for the LFO to gain overview of the large and continuous changing variety in referral options
Referral options from the LFO	Accessibility of referral options	Geographical accessibility	F	Health & fitness centres have nationwide coverage
			F	Care sport connector can be intermediary for searching appropriate referral option locally
			B	Organization of care sport connectors is different, making them harder to find
			B	Job description of care sport connector is different which makes it harder to use them as intermediary (not all are suitable)
Referral options from the LFO	Quality of refferal options	-	F	Create a network of quality partners
			B	Lifestyle coaches specialized in complex medical patients may not always be present in the field of sports and exercise
			B	Some lifestyle coaches do not have medical background

			B	Fitness industry feels that primary and secondary care do not see them as quality partners
			F	Health & fitness centres feel qualified to coach patients with different health conditions
Collaboration between LFO and CBLI	Referral content and process	-	B	Lifestyle Professionals believe that patients do not make an appointment with the CBLI on their own
			B	Making the appointment with the CBLI for patient, is missing an opportunity for enhancing self- management
			F	Relevant medical information in the referral about the patient is helpful for the CBLI
			B	Most CBLI are not primarily medically educated to deal with complex health problems
			F	Lifestyle professionals feel that the collaboration between LFO and CBLI is a shared task
Collaboration between LFO and CBLI	Communication platform	-	B	Secure email does not work between hospital and primary care
			B	Without secure communication it is not possible to give feedback of the patients progress
			F	There are a lot of communication systems in use with the CBLI
			B	The use of different systems is confusing for the CBLI
			B	Costs of paid communication systems is too high for the CBLI
Collaboration between LFO and CBLI	Partnership	-	F	CBLI is eager to work with the LFO
			B	Primary and secondary care is segregated
			F	Personal relationship is important for collaboration
			F	Being part of a network and working on the same case
