# Supplementary material for: Implementation barriers and facilitators for referral from the hospital to community-based lifestyle interventions from the perspective of lifestyle professionals: A qualitative study
Source: PLoS One. 2024 Jun 27;19(6):e0304053. doi: 10.1371/journal.pone.0304053 (PMC11210764; doi:10.1371/journal.pone.0304053)
Supplement: S4 Appendix — (PDF) [file pone.0304053.s004.pdf]

S4 Appendix. **Overview barriers, CFIR constructs, ERIC strategies and LOFIT strategies.**

Barrier	CFIR constructs	Eric strategies	LOFIT strategies
Lifestyle interventions can be too expensive for patients with low income	Available Resources, Costs	Access new funding	Identify benefits/arrangements for residents for patients with low income
		Fund and contract for clinical innovation	-
		Alter incentive/allowance structures	-
		Develop resource sharing agreements	Identify low cost and free referral options
Health care insurance coverage is not enough for needed care	External Policy & Incentives	Alter incentive/allowance structures	-
		Involve executive boards	-
		Build a coalition	-
		Capture and share local knowledge	Identify low cost and free referral options;<br>Organize lifestyle broker intervision to share experiences regarding referral options.
Applying for funding is difficult for some patients	External Policy & Incentives; Complexity	Capture and share local knowledge	Identify pathways of applying for funding; Create a network of professionals that can assist with applying for funding
		Identify and prepare champions	Develop informational material (i.e. video, flyer) to inform patients on how to apply for funding together with target population.
		Develop a formal implementation blueprint	-
		Create a learning collaborative	-
Few referral options for people who do not speak English or Dutch/Combined lifestyle intervention offered almost exclusively in Dutch language	Adaptability	Promote adaptability	Identify providers of interventions in different languages.
		Capture and share local knowledge	-
		Conduct local needs assessment	Conduct a needs assessment for identification of language adaptation needs
		Tailor strategies	-
It is hard for the LFO to gain overview of the large and continuous changing variety in referral options	Access to knowledge and information; Cosmopolitism	Conduct educational meetings	-
		Create a learning collaborative	Facilitate multidisciplinary meetings between the LFO and lifestyle professionals in the community
		Build a coalition	Build a network of/with referral options
		Develop educational materials	Develop a referral tool to find qualified lifestyle professionals in the patient's residential area
Care sport connectors are employed by different		Assess for readiness and identify barriers and facilitators	Interact with umbrella organizations and CSC businesses to discuss findability of CSC.

types of organizations which hampers the findability of the care sport connector	Structural characteristics, compatability	Promote adaptability	-
		Conduct cyclical small tests of change	-
		Tailor strategies	-
		Conduct local consensus discussions	-
Job description of care sport connector is different which makes it harder to use them as intermediary (not all are suitable)	Structural characteristics	Assess for readiness and identify barriers and facilitators	Identify different job profiles for CSC and develop a decision tree for referral.
		Change physical structure and equipment	-
		Build a coalition	-
		Identify and prepare champions	Include representatives of the care sport connectors in the development and implementation team.
Lifestyle coaches specialized in complex medical patients may not always be present in the field of exercise	Adaptability; Available resources	Access new funding	-
		Promote adaptability	Identify medical specialization of the referral options.
		Capture and share local knowledge	Identify conditions on when medical knowledge is needed and how this knowledge can be acquired
		Change physical structure and equipment	Add medical and safety information to the handover
Some lifestyle coaches do not have medical background	External policy & incentives	Alter incentive/allowance structures	Referral to professionals with a quality hallmark
		Involve executive boards	-
		Build a coalition	Identify referal options with a quality hallmark
		Capture and share local knowledge	Organise educational meetings for health care professionals in the community; Add medical and safety information to the handover
Fitness industry feels that primary and secondary care do not see them as quality partners	Culture; Learning climate	Facilitation	-
		Identify and prepare champions	Identify competencies of health & fitness centres
		Recruit, designate and train for leadership	-
		Assess for readiness and identify barriers and facilitators	Organise working visits to referral options
Lifestyle Professionals believe that patients do not make an appointment with the CBLI on their own	Compatibility; Executing	Identify and prepare champions	-
		Assess for readiness and identify barriers and facilitators	-
		Purposely reexamine the implementation	Discuss responsibility of patients regarding referral and offer assistance when necessary
		Conduct educational meetings	-

Secure email does not work between hospital and primary care/Without secure communication it is not possible to give feedback of the patients progress	Compatibility; Available resources	Access new funding	-
		Change physical structure and equipment	Use a secure network communication platform for handover; Discuss preferred ways of communication with referral option
		Conduct cyclical small tests of change	-
		Fund and contract for clinical innovation	-
The use of different systems is confusing for the CBLI	Complexity; Compatibility; individual stage of change	Promote adaptability	Develop a manual for the use of a network communication platform; Make lifestyle broker promote the digital platform with the referral partners
		Identify and prepare champions	Identify regional and national trends regarding network communication platforms
		Conduct cyclical small tests of change	-
		Assess for readiness and identify barriers and facilitators	-
Costs of paid communication systems is too high for the CBLI	Cost	Access new funding	-
		Alter incentive/allowance structures	Use network communication platforms without costs for other partners
		Develop resource sharing agreements	-
		Make billing easier	-
Primary and secondary care is segregated	Cosmopolitanism; compatibility	Build a coalition	Involve basic, primary and secondary care partners in development and implementation of the LFO and discuss how to avoid segregation locally
		Conduct local consensus discussions	Organize working visits to referral options; organize and/or participate in multidisciplinary meetings to connect care pathways
		Develop academic partnerships	-
		Promote network weaving	Build a network with CBLI representatives
