# Supplementary material for: Implementation barriers and facilitators for referral from the hospital to community-based lifestyle interventions from the perspective of lifestyle professionals: A qualitative study
Source: PLoS One. 2024 Jun 27;19(6):e0304053. doi: 10.1371/journal.pone.0304053 (PMC11210764; doi:10.1371/journal.pone.0304053)
Supplement: Attachment — Submitted filename: Response to reviewers.docx [file pone.0304053.s005.docx]

PONE-D-23-36328

Implementation barriers and facilitators for referral from the hospital to community-based lifestyle interventions from the perspective of lifestyle professionals: a qualitative study.

Dear Monika Blaszczyszyn,

Thank you for considering our manuscript entitled *Implementation barriers and facilitators for referral from the hospital to community-based lifestyle interventions from the perspective of lifestyle professionals: a qualitative study f*or publication in PLOSONE. We are grateful to you and to the reviewers for the time involved in reading our manuscript and for the helpful comments and suggestions to help us improve the manuscript and make the paper even stronger. Below we address the comments in a point by point response with a reference to page and line number in the revised manuscript with track changes.

Best regards,

Leonie te Loo

*Journal requirements:*

*1. When submitting your revision, we need you to address these additional requirements.*

*Please ensure that your manuscript meets PLOS ONE's style requirements, including those for file naming. The PLOS ONE style templates can be found at* 
[*https://journals.plos.org/plosone/s/file?id=wjVg/PLOSOne_formatting_sample_main_body.pdf*](https://journals.plos.org/plosone/s/file?id=wjVg/PLOSOne_formatting_sample_main_body.pdf) *and* 
[*https://journals.plos.org/plosone/s/file?id=ba62/PLOSOne_formatting_sample_title_authors_affiliations.pdf*](https://journals.plos.org/plosone/s/file?id=ba62/PLOSOne_formatting_sample_title_authors_affiliations.pdf)*.*

**Response #1:**

We checked the requirements and made minor changes to the file naming of the supplementary files.

*2. Thank you for stating the following financial disclosure: 
 [This research is part of the LOFIT-project which is funded by The Netherlands Organization for Health Research and Development (ZonMw), grant agreement no. 555003208. Also, the national knowledge and innovation network “Centre Of Expertise for Prevention in Care and Welfare” of the Inholland University of Applied Sciences (Hogeschool Inholland) funded this research.].  
Please state what role the funders took in the study.  If the funders had no role, please state: ""The funders had no role in study design, data collection and analysis, decision to publish, or preparation of the manuscript."" 
If this statement is not correct you must amend it as needed. 
Please include this amended Role of Funder statement in your cover letter; we will change the online submission form on your behalf.*

**Response #2:**

We added the role of the funders in the cover letter.

*3. We note that you have indicated that there are restrictions to data sharing for this study. PLOS only allows data to be available upon request if there are legal or ethical restrictions on sharing data publicly. For more information on unacceptable data access restrictions, please see* [*http://journals.plos.org/plosone/s/data-availability#loc-unacceptable-data-access-restrictions*](http://journals.plos.org/plosone/s/data-availability#loc-unacceptable-data-access-restrictions)*.*

*Before we proceed with your manuscript, please address the following prompts:*

*a) If there are ethical or legal restrictions on sharing a de-identified data set, please explain them in detail (e.g., data contain potentially identifying or sensitive patient information, data are owned by a third-party organization, etc.) and who has imposed them (e.g., a Research Ethics Committee or Institutional Review Board, etc.). Please also provide contact information for a data access committee, ethics committee, or other institutional body to which data requests may be sent.*

*b) If there are no restrictions, please upload the minimal anonymized data set necessary to replicate your study findings to a stable, public repository and provide us with the relevant URLs, DOIs, or accession numbers. For a list of recommended repositories, please see*
[*https://journals.plos.org/plosone/s/recommended-repositories*](https://journals.plos.org/plosone/s/recommended-repositories)*. You also have the option of uploading the data as Supporting Information files, but we would recommend depositing data directly to a data repository if possible.*

*We will update your Data Availability statement on your behalf to reflect the information you provide.*

**Response #3:**

All relevant data are within the manuscript and its Supporting Information files. Within Amsterdam UMC it is mandatory to delete audio recordings after transcribing and checking. The (Dutch) transcripts of the interviews, are available upon reasonable request due to ethical and legal restrictions under the General Data Protection Regulation (EU). The study participants did not consent to make the full transcripts of interviews publicly available. Excerpts of the raw data (quotes) are available within the text of this paper. Requests for anonymized excerpts from the full transcripts can be send to the general email address: lofit@amsterdamumc.nl

*4. One of the noted authors is consortium [LOFIT-consortium]. In addition to naming the author group, please list the individual authors and affiliations within this group in the acknowledgments section of your manuscript. Please also indicate clearly a lead author for this group along with a contact email address.*

**Response #4:**

in the acknowledgement section the individual authors of the consortium are listed. We added their affiliations and indicated a lead author and email address for the consortium on page 34-35, lines 690-724.

*5. We note that Figure 1 in your submission contain copyrighted images. All PLOS content is published under the Creative Commons Attribution License (CC BY 4.0), which means that the manuscript, images, and Supporting Information files will be freely available online, and any third party is permitted to access, download, copy, distribute, and use these materials in any way, even commercially, with proper attribution. For more information, see our copyright guidelines:* [*http://journals.plos.org/plosone/s/licenses-and-copyright*](http://journals.plos.org/plosone/s/licenses-and-copyright)*.
 
We require you to either (1) present written permission from the copyright holder to publish these figures specifically under the CC BY 4.0 license, or (2) remove the figures from your submission:
 
a. You may seek permission from the original copyright holder of Figure 1 to publish the content specifically under the CC BY 4.0 license. 
 
We recommend that you contact the original copyright holder with the Content Permission Form (*[*http://journals.plos.org/plosone/s/file?id=7c09/content-permission-form.pdf*](http://journals.plos.org/plosone/s/file?id=7c09/content-permission-form.pdf)*) and the following text:
“I request permission for the open-access journal PLOS ONE to publish XXX under the Creative Commons Attribution License (CCAL) CC BY 4.0 (*[*http://creativecommons.org/licenses/by/4.0/*](http://creativecommons.org/licenses/by/4.0/)*). Please be aware that this license allows unrestricted use and distribution, even commercially, by third parties. Please reply and provide explicit written permission to publish XXX under a CC BY license and complete the attached form.”
 
Please upload the completed Content Permission Form or other proof of granted permissions as an ""Other"" file with your submission. 
 
In the figure caption of the copyrighted figure, please include the following text: “Reprinted from [ref] under a CC BY license, with permission from [name of publisher], original copyright [original copyright year].”
 
b. If you are unable to obtain permission from the original copyright holder to publish these figures under the CC BY 4.0 license or if the copyright holder’s requirements are incompatible with the CC BY 4.0 license, please either i) remove the figure or ii) supply a replacement figure that complies with the CC BY 4.0 license. Please check copyright information on all replacement figures and update the figure caption with source information. If applicable, please specify in the figure caption text when a figure is similar but not identical to the original image and is therefore for illustrative purposes only.*

**Response #5:** we have altered the figure so that it complies with the CC BY 4.0 license. The current images that are in the figure have been made especially for our study.

*Reviewer #1: Comments to author


Abstract
Under the method section, avoid using abbreviations or provide the full name (CFIR, ERIC) for those unfamiliar with them.*

**Response #6**:

Thanks for this solid advice. We have provided the full names of the abbreviations (the consolidated framework for implementation research (CFIR) in combination with the Expert Recommendations for Implementing Change (ERIC) strategies) in the revised manuscript with track changes on page 3 , line 51-53.

*Introduction

Line 87: consider providing some additional information about the I-change model. It would be helpful for the readers to understand what this model is about.*

**Response #7**:

We named the I-Change model to support the idea that different interacting factors lead to health behavior. This model is one of many and because we did not use it further in the study, we can see that it is confusing to just name this one. Because of this we removed the name of the model in the text on page 5, line 92; which is in agreement with the advice of reviewer 2 as well.

*Line 90: you mentioned that lifestyle coaching in the community is less costly. To whom? To the patients or the healthcare system. If it is less costly for the healthcare system, do you need to hire LFO staff? How many? Do you need to implement a certain referral system? Do you need a clinic to meet with the patients? Is it not costly to implement such a service considering all the mentioned resources?
Is there any published cost-effectiveness analysis to support your statement? Please provide further explanation. Also, it would be helpful to know when the service was implemented in the Netherlands. Especially since most of your participants, 61%, had a working experience of less than 5 years.*

**Response #8:**

What was meant here is that lifestyle coaching in the community is less costly for the healthcare system than lifestyle care in the hospital, due to the complexity of care in an UMC.

In the Netherlands health care (in the hospital and in the community) is financially almost completely covered by basic health insurance. Citizens are required to have basic health care insurance for which they pay a monthly contribution. When patients use care, they additionally pay a maximized yearly excess. The Dutch government aims to keep this system affordable by designating care at the best quality-cost ratio, which means that when possible, care is given in the community instead of the hospital. Ultimately this flows back to the patients as well, as expensive care will lead to higher basic health insurance contribution.

The current study served to develop the LFO, which was not yet existing at that time. Using the results of this study, we implemented the LFO and simultaneously started a (cost) effectiveness study. We agree with the reviewers that the manuscript does not clearly state that the LFO is a novel care concept that still had to be developed. To clarify this, we have made changes to the manuscript on page 3, line 37 ; page 6, line 102-104; page 7, line 121; page 8, lines 127-128 to make this clearer.

The reviewer rightly states that it can be costly to implement an LFO in a hospital, given the resources required. We don't yet know how expensive it is and whether it outweighs the benefits. At this moment, we are executing an RCT to establish the effectiveness and cost-effectiveness of the LFO. The RCT is still ongoing, therefore we have not yet published results.

*Line 99: you mentioned that the lifestyle broker has motivational interviewing skills. What is their qualification? E.g., a dietician or other medical specialty? Are there any required experiences?*
***The procedure of the referral process is not very clear,*** *how and where they discuss the patients needs? Do the patients need to get an appointment by themselves, or do they get referred by their physician to LFO?
Also, it is not clear where and how the patient will receive the CBLI intervention? At their homes, or particular community or primary care clinics? How would the CBLI communicate with the LFO? Do they provide any written progress notes/letters/emails to the LFO?
Please provide additional information for international readers unfamiliar with this service, as their understanding of the nature of the service will help them understand the subsequent sections.*

**Response #9**: Thank you for pointing out that this section needs clarification. We adjusted this section with more information on the procedure (page 6, lines 105-110). However, finding out how the exact procedure of the referral process should be, was part of this research; since we wanted to align as much as possible to the working processes and the preferences of the community-based lifestyle interventions (CBLI). We added this information on page 6, lines 118-120.

*Line 110: who are the stakeholders in this context? The patients, staff of the LFO, and CBLI? Any others? What about the service leaders or implementers? Did you consider interviewing them? If not, do you think this is one of the limitations? You did interview all the stakeholders. Is that a limitation?*

**Response #10**: We agree with the reviewer that for successful implementation we must include the perspectives of all stakeholders. In this study, we focused on the external stakeholders of the LFO: the CBLI. Our aim was to design our process and procedures in alignment with the views of these stakeholders. In another study, we focused on the internal stakeholders of the LFO: the health care professionals in the hospital and also the patients. We added this information in the discussion on page 32, line 664- 667. The research team was and is also responsible for the development and implementation of the LFO. In this way, we have taken into account the perspectives of all relevant stakeholders.

*Method
Line 114: “we performed semi-structured interviews with various CBLI”. I am confused with using the abbreviation CBLI, which refers to community-based lifestyle interventions, not personnel. I think it is better to use another abbreviation or term, e.g., community-based healthcare providers, and change it throughout the manuscript.*

**Response #11:** we agree, that is confusing. The CBLI are lifestyle interventions that the LFO can refer to. We interviewed professionals that work for organizations that provide these lifestyle interventions. To make it not more confusing by adding another new term and abbreviation, we decided to add the word ‘professional’ after CBLI wherever we are referring to the person and not to the intervention.

*Line 115-117: “Our aim was to explore views, needs and preferences regarding implementation and collaboration with an LFO in these two medical centers”. I prefer to mention your aim once in the introduction. This is a repetition, and you did not mention your participants here.*

**Response #12:** we agree. We removed the aim here**.**
 *Line 118-119: Why was the study exempted from the review by the ethical committee? you can add the statement from your supplementary file 5 here, “The research does not fall within the scope of the Medical Research Involving Human Subjects Act (WMO). The reason is that the research does not have a medical-scientific question”. And remove the file S5.*

**Response #13:** thanks for the suggestion, we did as such.  *Line 121: the provided Figure 1 is not of high quality, I could not read what is written in it. Please provide a higher-quality figure.*

**Response #14:** we changed the figure and provided a higher-quality figure

*Line 158-159: I do not understand what the BLCN stands for. Also, does GLI stand for Combined Lifestyle Intervention? Or is it CLI? Is it a typo error?*

**Response #15:** these were the Dutch abbreviations. We replaced them by English abbreviations: (Association for Lifestyle Coaches in the Netherlands (ALCN); Combined Lifestyle Intervention (CLI)) (page 10, line 178-180).

*Line 168: what is the necessity of doing a video call interview? Why not a phone call? Isn’t easier?*

**Response #16:** these interviews were held during the time that COVID-19 was limiting the possibility for physical meetings, which we would have preferred for the interviews in order to be able to fully engage with the respondent. A video call was in our view the best alternative to physical meetings. While telephone calls would have been possible and perhaps easier, we believed they would not have provided the same connection with, and in-depth information from, the respondents.

*Line 181: could be? Or some sections were skipped!*

**Response #17:** the reviewer is correct; some sections were skipped depending on the job title. We changed that (page 11, line 202). *Line 186: you introduce the service to the participants; this means that not all of them were familiar with the service. Should you clarify this in your inclusion criteria? E.g., participants were deemed eligible to participate whether they had previous knowledge of the service or not.*

**Response #18:** we recognize that we did not state clearly enough in the introduction that the service (LFO) did not yet exist at all. We hope that it is now much clearer due to the changes we made (see response #8, third paragraph).

*Line 202: Were the interviews in English? Or in Dutch, so did you have to translate them? If yes, what was the method of translation of the transcripts? If you did not translate the transcripts, does the used software support the Dutch language?*

**Response #19:** The interviews were in Dutch, the transcription, coding and analysis were all in Dutch, which was supported by the software we used. We ultimately translated the quotes we used in the manuscript. Author LL carefully made the translations and they were checked and if necessary improved by author JJ, whereafter they were discussed until consensus was reached.

We added in the manuscript that all interviews and analysis were in Dutch on page 11, line 209; page 12, lines 236-237.

*Line 224: you mentioned that translations were made at the end, does this mean all the previous steps were in Dutch?*

**Response #20:** See response #19. *Results
Line 241: it would also be helpful to know how many were already familiar with the referral service. They might have more insight into the barriers and facilitators if they witness the early implementation of the service, in which case they can explore barriers/facilitators at the early stage of the implementation and after running the service for several years.
The participant's knowledge of the service was in mind as I read through the quotations.*

**Response #21:** that is correct, as mentioned in response #8, the LFO service was not yet existing.

*Line 414: Is the communication between 1ry and 2ry care always via email? Is there any paper referral? I think you need to better explain the service in the introduction, so when the reader comes to this section, he/she will already have enough understanding of the procedure.*

**Response #22:** in the Netherlands it is common to communicate mostly via (secured) email and not via paper referral anymore. In this research we wanted to find out how the CBLI-professionals wanted to communicate with the LFO and what their experiences were (regarding communication) with other organizations or with other departments within the hospital, see response #8. No further additions were made.

*You did not mention when you reached the saturation, and what types of saturation was considered in this study? What about triangulation, credibility, and transferability of the data.*

**Response #23:** we considered thematic saturation; it is now added in the method (page 9, line 161; page 12, line 219) with a reference to:

21.	Saunders B, Sim J, Kingstone T, Baker S, Waterfield J, Bartlam B, et al. Saturation in qualitative research: exploring its conceptualization and operationalization. Qual Quant. 2018;52(4):1893-907

We added information about triangulation, credibility and transferability of data to the strength and limitations section of the discussion on page 31, line 642 and page 31-32, lines 652-656.

*Elaborate more on why you choose interview vs. focus group. Did you reach the data saturation? For some professions, you only interviewed 1, like a dietician; how did you reach saturation about the perspectives of this professional? Could consider this in the limitation of the study?*

**Response #24:** the choice for interviews instead of focus groups was pragmatic, given the expected time constraints of the professionals. We added an explanation on the choice of interviews on page 8 in line 130-132.

It is correct that we only interviewed one dietician, it is possible that if we had interviewed other dieticians, we would have had richer data. However, we interviewed all respondents in the role they (will) play as CBLI-professional in relation to the LFO and not on their specific expert knowledge. We feel therefore that it is not a limitation of this study. We added this on page 31, line 646-648.

*Discussion
Line 582: is the abbreviation correct? Integral Health care Agreement (IZA)?*

**Response #25:** This is de Dutch abbreviation (of “Integraal Zorg Akkoord”) which does not have a formal English abbreviation. Also, in the English translation of the agreement this abbreviation is used, which is why we did not change it, but added that this is the Dutch abbreviation on page 31 in line 636.

*Line 588: maybe you can discuss the triangulation here “A strength of this study is the input from various lifestyle professionals”*

**Response #26**: thank you for this suggestion, which we followed (page 31, line 642).

*Line 596-597: “The interviews were performed in order to develop the LFO”. It would be better to move this to the introduction. Because the information provided in the introduction gives the reader the impression that the service is already available but partially implemented. This need to be clarified in the introduction and your aim.
Line 598: Did none of the participants have any experience with the service?*

**Response #27**: see response #8, third paragraph.

*Line 599: I appreciate that you used evidence-based tools to develop your strategies and map them with the CFIR framework, but I was expecting to read some examples from previous studies showing that these strategies did work in practice. You did not mention anything about the improvement strategies in the discussion section.

What are the practical implications or recommendations for practice? You may need to write a few lines about it.*

**Response #28:** Previous studies have evaluated CFIR-ERIC -based implementation strategies to real world strategy-effectiveness, we added the following reference on page 29, line 596.

27.	Yakovchenko V, Lamorte C, Chinman MJ, Goodrich DE, Gibson S, Park A, et al. Comparing the CFIR-ERIC matching tool recommendations to real-world strategy effectiveness data: a mixed-methods study in the Veterans Health Administration. Implementation Science. 2023;18(1)

Furthermore, we are aware of the theoretical base of current strategies and the need for evaluating the implementation strategies which we will do in the process evaluation of the LOFIT study. We added this information on page 32-33, line 675-679.

*Conclusion
The conclusion is not firm and needs some work. You need to discuss the implications for practice and some future work to strengthen your conclusion.*

**Response #29:** we enhanced the conclusion by making the implications more specific on page 32, line 673-675 and also by referring to our future research work in which we will evaluate the strategies, on page 32-33, line 675-679.

*Reviewer #2: 

In the abstract results, this is not clear: 'determine qualifications professionals'. Please revise.*

**Response #30:** thank you for your detailed feedback and for bringing this to our attention. We added the term “lifestyle” to make clear which professionals we are talking about on page 4, line 62 and page 24 line 503 and on page 24 in Table 2**.**

*In the introduction: 'as patients are more motivated for a
82 lifestyle change when experiencing a health event.' Do the authors have any evidence to support this?*

**Response #31:** Yes, we added the following three references which support this statement on page 5, line 87.

5.	Tofler GH, May R, Bartrop R, Kirkness A, Glinatsis H, de Burgh S. Acute Coronary Syndrome as a Teachable Moment for Smoking Cessation. J Smok Cess. 2015;10(1):5-11.

6.	McBride CM, Emmons KM, Lipkus IM. Understanding the potential of teachable moments: the case of smoking cessation. Health Educ Res. 2003;18(2):156-70.

7.	Brust M, Gebhardt WA, Numans ME, Kiefte-de Jong JC. [Teachable moments: the right moment to make patients change their lifestyle]. Ned Tijdschr Geneeskd. 2020;164.

*The authors make reference to the I-Change model but give no explanation of it and its significance. I would recommend providing a brief outline or removing the mention of this.*

**Response #32***:* The I-Change model was intended as an example of a model that describes health behavior, but has no specific function. Therefor we have removed the mention of this. See response #7

*The LFO appears to be a novel concept. Is this just being implemented in the researchers' area or is this a national model. It would be good to provide a little bit of information about where this model is being provided and how successful this is if evidence exists.*

**Response #33:** At the time of this study the LFO was a novel concept (it did not exist and was not implemented) and as such we explored implementation barriers and facilitators from a community perspective (this study). The results of the current study informed the further development of the LFO. We are currently investigating the effectiveness and cost-effectiveness of the LFO (= LOFIT project)

We made more clear in the manuscript that this model did not exist yet. See response # 8, third paragraph.

*Please fix the first line in the table where it states 'total = 23242'*

**Response #34:** Resolved.

*It seems the themes might be better labelled:
'LFO knowledge about financial status and/or health eligibility of the patient'
'LFO knowledge about the cultural and linguistic needs of the patient'
'LFO knowledge about the structure and availability of CBLI services'
'LFO knowledge about the quality of CBLI services'
'Agreed referral process and content'
'Secure and equitable communication platform'
'Personal relationships between the LFO and CBLI to facilitate collaboration'*

**Response #35:** First of all, we thank you very much for the effort you made here to advise us in detail. We feel however, that we cannot change these. During analysis, as a research team we discussed the barriers and facilitators and decided together that a pragmatic arrangement according to the workflow in the LFO process would be most fitting to present the results: in the LFO, first the patient and lifestyle broker have to find a suiting referral option (which is hindered by mentioned barriers) and after that, the LFO will need to collaborate with the CBLI. The barriers and facilitators represent the views of the CBLI-professionals and not that of the LFO.

*Develop support tools: how should the barrier about keeping up to date with appropriate services available be overcome?
Would a manual about the use of the communication tool be the best option? Would it not be better to find a system-wide digital solution to facilitate easy communication?*

**Response #36**: due to your questions we recognize that we did not make it clear enough which strategy links to which barrier (e.g. *the manual of the communication tool* is a proposed solution for the barrier: *The use of different systems is confusing for the CBLI* and we suggest *making a* *referral tool* to overcome the barrier: *It is hard for the LFO to gain overview of the large and continuous changing variety in referral options*).
Therefore, on page 25-28 , in the text below table 2 we made this more insightful by adding information on which barrier the specific strategy links to. We agree with the reviewer that a system-wide digital solution would be the best strategy, this is what we mean with the proposed (digital) communication platform. The word “digital” on page 26 in line 531 and 538 was added.

*Build networks: this is really vague. I recommend the authors reconsider what the network could look like, how this might be established and sustained.*

**Response #37:** The use of networks is twofold: (1) to keep up with possible referral options and (2) to improve collaborating within and between care pathways. We expended this section in the manuscript and gave some specific examples on page 27 , lines 542-550.

*Facilitate learning collaboratives: these strategies appear to present a significant workload for the LFO. Can the authors potentially consider other options that might be more feasible and attractive?*

**Response #38:** We agree with the reviewer that it is a significant workload. We also expect that it is a necessary one, since the LFO works together with many different parties in the hospital as well as the community, all with different education, knowledge and skills. The proposed implementation strategies will be evaluated in future research, see response #29.

*Please can the authors clarify this sentence:' In order to realize this, representatives of the care sport connectors could be included in the development and implementation team before implementation. '*

**Response #39:** We found that the supply of referral options in the field of exercise and sports is so large, that a local intermediate (a care sport connector) who knows the referral options in his specific region, may be very helpful for the referral process. How to deploy this intermediate in the referral process, is not known and we need a representative/coordinator of the care sport connecters to think along with the research team/ lifestyle brokers on how this should be done and what agreements must be made.

The line is rewritten as following: **Representatives of the care sport connectors should be included in the development before the start of the LFO, to think along with the implementation team on how this should be done and what agreements must be made** (page 28 line 568-571).

*The key concern with the ERIC strategies is that they are proposed by the research team. Not all seem to be very practical or feasible. It is not clear if the research team have sufficient knowledge and expertise to add credibility to recommending these strategies. Because of this, the recommendations have little value.*

**Response #40:** We feel that we have not explained the development of the strategies and our role in the implementation process well enough. We have made alterations in the text on page 13, lines 245-250.

*Discussion: I would argue that the limited number of referral options for low income patients and for non-Dutch speaking people and those living in rural areas and quality of referral options are not barriers for the implementation of a hospital based LFO. These are simply limitations of the current CBLI offer...*

**Response #41**: we partly agree; they are indeed limitations of the current CBLI offer. However, they do negatively influence the process of the LFO and thereby is a barrier for the LFO. If people cannot be referred to a CBLI because there is none available, the patient is not helped. For the implementation of the LFO we needed to identify this barrier in order to be able to try to solve it.

*What do the authors mean here: '. Matching strategies were developed and for the
larger part implemented. '?*

**Response #42:** We agree, of course at the time we had not implemented the strategies.

The line is rewritten as following: **We used the CFIR-ERIC-tool to select effective, actionable strategies [27], which we operationalized into practical strategies for the LFO.** Page 29, lines 595-597.

*How do the authors know that these were the most important strategies: ' Most important strategies concerned identifying limited referral options and professionals with a quality hallmark and strategies to streamline the actual referral and the process of collaboration between basic services, primary and secondary
care*

**Response #43:** We agree, of course at the time we could not have known what would be the most important strategy.

The line is rewritten as following: **Strategies concerned identifying limited referral options and professionals with a quality hallmark and strategies to streamline the actual referral and the process of collaboration between basic services, primary and secondary care**. Page 29, lines 597-600.

*What is the relevance of this in the context of this study: ' Digital health can facilitate and improve health care in various applications, such as
diagnostic tools, in order to make disease management and communication health care
processes more patient-centered [44, 45].'*

**Response #44:** the relevance here was discuss the importance of digitization in health care, which is part of some of the implementation strategies. We agree that ‘diagnostic tools’ might be less relevant.

This line is rewritten as following: **Digital health can facilitate and improve health care in various applications in order to make disease management and communication in the health care processes more patient-centered**. Page 31, lines 631-633.

*The reader becomes aware: 'The interviews were performed in order to develop the LFO.' In the last section of the discussion! This should be made clearer at the beginning, when rationalising the need for the study.*

**Response #45:** yes, we agree absolutely, that this was not clear enough. We made changes to improve this all over the manuscript. See response #8, third paragraph. (Changes on page 3, line 37 ; page 6, line 102-104; page 7, line 121; page 8, lines 127-128).

*I do not agree with this strength: ' A major strength of this study is that we systematically matched
600 evidence-based implementation strategies to the found barriers of the CBLI.' Given how these strategies were selected, I am unconvinced that these are the most practical, feasible and cost effective recommendations to implement the LFO.*

**Response #46:** in future research, we will evaluate the strategies to be able to draw conclusions about their effectiveness. See also response #28.

*Given this paper is about developing an intervention to be provided at the intersection between primary and secondary care, the discussion should be considering wider literature about other interventions that have been developed and tested at this juncture, e.g. care coordinators, discharge planners. disease-specific interventions if appropriate.*

**Response #47:** Thank you for the suggestion. We added a few lines about this in the discussion on page 30, lines 626-630
